# Adverse childhood experiences and prescription opioid use during pregnancy: an analysis of the North and South Dakota PRAMS, 2019–2020

**DOI:** 10.1186/s12884-023-05925-7

**Published:** 2023-08-23

**Authors:** Alexander Testa, Benjamin Jacobs, Lixia Zhang, Dylan B. Jackson, Kyle T. Ganson, Jason M. Nagata

**Affiliations:** 1 Department of Management, Policy and Community Health, University of Texas Health Science Center at Houston, Houston, USA; 2 Burnett School of Medicine at TCU, Texas Christian University, Fort Worth, USA; 3 Raymond A. Kent School of Social Work and Family Science, University of Louisville, Louisville, USA; 4 Johns Hopkins Bloomberg School of Public Health, Johns Hopkins University, Baltimore, USA; 5 Factor-Inwentash Faculty of Social Work, University of Toronto, Toronto, Canada; 6 Department of Pediatrics, University of California, 550 16th Street, Box 0503, San Francisco, CA 94158 USA

**Keywords:** Pregnancy, Adverse childhood experiences, Prescription opioid, PRAMS

## Abstract

**Objectives:**

This study assesses the association between adverse childhood experiences (ACEs) and prescription opioid use during pregnancy.

**Methods:**

This study uses data on 2,999 individuals from the 2019 and 2020 Pregnancy Risk Assessment Monitoring System (PRAMS) from North Dakota and South Dakota. The relationship between ACEs and prescription opioid use during pregnancy is examined using multiple logistic regression.

**Results:**

The prevalence of prescription opioid use increases alongside more ACE exposure. Compared to those with no ACEs, recent mothers with three or more ACEs have a 2.4 greater odds of prescription opioid use during pregnancy (aOR [adjusted odds ratio] = 2.437; 95% CI [confidence interval] = 1.319, 4.503).

**Conclusion:**

Exposure to three or more ACEs are associated with a higherrisk of prescription opioid use during pregnancy. Additional research is needed better understand the mechanisms that link ACEs and prescription opioid use during pregnancy, as well as how to best support those with ACEs exposure in a trauma-informed manner to reduce the risk of substance use.

**Supplementary Information:**

The online version contains supplementary material available at 10.1186/s12884-023-05925-7.

## Introduction

From 2020 to 2021, over 107,000 drug overdoses occurred in the United States [1]. A substantial driver of overdose mortality is opioids, which accounted for approximately 76% of all drug overdose deaths in 2021 [1]. The opioid epidemic has touched many segments of the population over the past two decades. However, during the opioid epidemic crisis, pregnant women are a important population of focus [2].

Indeed, prior research demonstrates a relationship between prescription opioid analgesics use (i.e., butorphanol, buprenorphine for pain, codeine, fentanyl, hydrocodone, meperidine, methadone for pain, morphine, opium, oxycodone, pentazocine, tapentadol, and tramadol) and adverse infant birth outcomes including poor fetal growth, preterm birth, congenital disabilities, and neonatal abstinence syndrome [3–7]. Despite the risk prescription opioids pose for maternal and infant health, estimates suggest that nearly seven percent of women have reported using a prescription opioid during pregnancy, and among these, 21.2% reported misuse (i.e., obtaining from a source other than a health care provider or using for a reason other than pain) [8]. Given the potential harms of prenatal prescription opioid exposure to offspring’s health and development, it is essential to identify the factors associated with prenatal prescription opioid use to inform policy and practice better. Of notable importance is the role of earlier stressful life events in contributing to the risk of prescription opioid use during pregnancy [9].

Adverse childhood experiences (ACEs) are experiences with abuse, neglect, and household dysfunction during childhood and adolescence [10]. Research on ACEs demonstrates that they are highly powerful, with exposure to ACEs increasing the chance of poor outcomes later in life, including negative physical and mental health [11, 12], diminished employment prospects [13], barriers to health care utilization [14], and premature mortality [15]. In particular, ACEs have a dose–response relationship with unwanted outcomes such that experiencing more ACEs—such as three or more—is associated with worse outcomes [16]. Prior research finds that ACEs are associated with substance use in adulthood [17–20], including both prescription and illicit opioid use [21–24]. However, limited research has investigated the relationship between ACEs and prescription opioid use during pregnancy, despite extant research documenting a connection between ACEs and other types of substance use during pregnancy, including various illicit drugs [25, 26], tobacco [26], alcohol [27], marijuana [28, 29]. One study on the relationship between ACEs and prescription opioid use in pregnancy uses an unrepresentative sample of 303 pregnant women in a psychosocial perinatal support program in a Southern urban medical clinic, finding no association between more ACEs exposure and prescription opioid use for nonmedical reasons during pregnancy [30], although certain ACEs subtypes such as being exposed to childhood maltreatment (i.e., emotional abuse, physical abuse sexual abuse, emotional neglect, and physical neglect) were associated with prescription opioid use during pregnancy.

Using data on representative samples of live births in two U.S. states, the current study extends prior literature by examining whether ACEs exposure is associated with prescription opioid use during pregnancy.

## Methods

### Data

Data are from the Pregnancy Risk Assessment Monitoring System (PRAMS). The PRAMS is an ongoing population surveillance system of live births in the United States conducted by the Centers for Disease Control and Prevention (CDC) and state health departments. Data are collected yearly via a stratified systematic sample of birth certificate records. The PRAMS data are from three separate sources: (1) birth certificate records, (2) vital record systems, and (3) responses to a PRAMS survey. The PRAMS survey is mailed to the home address of recent mothers approximately 2 to 4 months following their birth delivery. After up to three mailing attempts, telephone calls are made to non-responders. Survey weights enable adjustment for non-response and non-coverage, thereby making samples representative of live births in a given state [31].

While the entire PRAMS collects data from 46 states representing approximately 81% of all U.S. live births, a subset of states in specific years ask topic-specific questions. In 2019 and 2020, a supplemental survey was administered to a subset of jurisdictions asking about prescription opioid use during pregnancy [8]. In addition, select states include topic-specific questions asking mothers about various life experiences. Only two states—North Dakota and South Dakota—have questions asking about mothers’ adverse childhood experiences [32]. Accordingly, the current study uses data on 2,999 mothers from the 2019 and 2020 PRAMS surveys conducted in North and South Dakota. Additional file 2: Appendix A provides a flow chart describing the analytic sample section.

The study was performed in accordance with the Declaration of Helsinki. The study was approved by the Centers for Disease Control and Prevention in accordance with the data usage agreement for the Pregnancy Risk Assessment Monitoring System. All participants provided informed consent; for minors younger than 18 informed consent was waived by CDC. The general PRAMS methodology and protocol have been reviewed and approved by the CDC institutional review board, and state PRAMS projects undergo review by the local institutional review board of record for the health department. An informed consent document in each survey packet explains a participant’s rights in mail surveys. No written consent is required; consent is implied if the survey is completed and returned [31]. The informed consent document is read verbally for phone interviews, and the participant verbally agrees to proceed with the survey. Minors younger than 18 years who have given birth are considered emancipated for decisions about their children and do not require consent from parents or guardians to participate [31]. The PRAMS survey is administered in both English and Spanish. While an interviewer verbally administers phone-based surveys, mail surveys may depend on a women’s literacy levels and comfort in responding to the survey, neither of which are assessed in the PRAMS data collection process, and therefore consent is not requested from a parent or legal guardian in cases when an individual has low literacy levels [33].

### Dependent variable

Consistent with prior research [8, 9, 34], *any prescription opioid use* is a dichotomous indicator of whether a respondent reported using any prescription opioids during their most recent pregnancy. Respondents were asked, “During your most recent pregnancy, did you use any of the following prescription pain relievers?”: (a) hydrocodone (like Vicodin®, Norco®, or Lortab®), (b) codeine (like Tylenol® #3 or #4, not regular Tylenol®), (c) oxycodone (like Percocet®, Percodan®, OxyContin®, or Ultracet®), (d) tramadol (like Ultram® or Ultracet®), (e) hydromorphone or morpheridine (like Demorol®, Exalgo®, or Dilaudid®), (f) oxymorphone (like Opana®), (g) morphine (like MS Contin®, Avinza® or Kadian®), or (h) fentanyl (like Duragesic®, Fentora®, or Actiq®). Respondents who answered affirmatively to using any of these prescription opioids during pregnancy were coded as a value of 1; those who did not indicate any use of these prescription opioids were coded as 0.

### Independent variable

ACEs were measured using respondent self-report on ten types of childhood adversity before age 18. The ten questions used to classify ACEs closely approximate the measures from the CDC-Kaiser ACE Study [10]. Additional file 2: Appendix B presents the definitions and prevalence for the ten items. Consistent with prior research using PRAMS data, responses to the 10 ACE items are combined to create a cumulative score ranging from 0–10. The total ACEs scores were grouped into four categories: 0 ACE, 1 ACE, 2 ACEs, 3 or more ACEs [9, 32].

### Control variables

Control variables include the mother’s age (< 24, 25–29, 30–34, and 35 or older), mother’s race/ethnicity (White, Hispanic, Black, Native American, Asian/Other, and mixed race), mother’s educational attainment (0 = less than college, 1 = college graduate), marital status (0 = not currently married, 1 = currently married), number of prior births (0, 1, 2, or 3 +), whether a mother reported being on Medicaid in the three months before pregnancy (1 = yes; 0 = no), household income (≤ $16,000, $16,000-$40,000, $40,001-$85,000, or > $85,000), state of residence, and year of birth.

### Analytic approach

The bivariate association between the number of ACEs and prescription opioid use during pregnancy is assessed using a chi-square (χ^2^) test. Multivariable logistic regression is used to examine the associations between ACEs exposure (0, 1, 2, or 3 + ACEs) and prescription opioid use during pregnancy, net control variables. Analyses also assess the relationship between exposure to each specific type of ACE and prescription opioid use during pregnancy. All data analyses were conducted using the *svy*package for weighted survey data in Stata/S.E. version 17. Variance inflation factors were under 2, indicating no significant issues with multicollinearity [35].

## Results

**Fig. 1:**
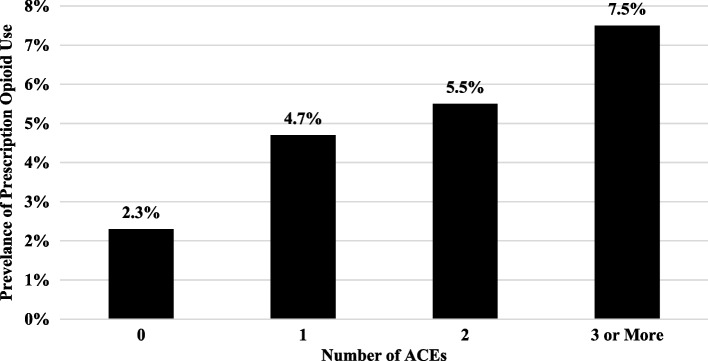
Prevalence of prescription opioid use during pregnancy by number of Adverse Childhood Experiences (ACEs). *Note*: Results of a χ^2^ with 3 degrees of freedom shows a statistically significant difference between prescription opioid use during pregnancy across the number of ACEs (χ.^2^ = 37.69, *p* < .001)

Summary statistics are presented in Table 1. Overall, 4.7% of the sample reported prescription opioid use during pregnancy; 39.5% reported no ACEs, and 30% of the sample reported three or more ACEs. Figure 1 shows that the prevalence of prescription opioid use increased alongside higher ACEs score: 0 ACEs (2.3%), 1 ACE (4.7%), 2 ACEs (5.5%), and 3 or more ACEs (7.5%). A chi-square test reveals a statistically significant difference between the prevalence of prescription opioid use in different ACEs groups (χ^2^ = 37.69, *p* < 0.001).

**Table 1:** Weighted summary statistics of analytic sample (*N* = 2,999)

Variable	%
Prescription Opioid Use	4.7%
*Number of ACEs*	
0	39.5%
1	19.0%
2	11.5%
3 or More	30.0%
*Mother’s Age*	
Less than 24	20.0%
25–29	34.3%
30–34	31.7%
35 or Older	13.9%
*Mother’s Race/Ethnicity*	
White	75.1%
Hispanic	4.8%
Black	4.4%
Native American	9.7%
Asian/Other	2.5%
Mixed Race	3.4%
*Mother’s Educational Attainment*	
Less than High School	9.2%
High School Graduate	20.7%
Some College	30.4%
College Graduate	39.8%
Currently Married	68.5%
*Number of Prior Births*	
0	34.9%
1	31.2%
2	18.8%
3 +	15.0%
Medicaid	12.9%
*Household Income*	
≤ $16,000	15.0%
$16,000-$40,000	17.7%
$40,001 – $85,000	36.5%
> $85,000	30.9%
*State of Residence*	
North Dakota	43.4%
South Dakota	56.6%

*Abbreviations: ACEs* Adverse childhood experiences

The results of the multivariable logistic regression in Table 2 show that compared to those with no ACEs, recent mothers with three or more ACEs had approximately a 2.4 greater odds of prescription opioid use during pregnancy (aOR [adjusted odds ratio] = 2.437; 95% CI [confidence interval] = 1.319, 4.503). The multiple logistic regression analysis Additional file 2: Appendix C detail that eight of the 10 ACEs (except for parental separation and household violence) had a positive and statistically significant association with prescription opioid use during pregnancy.

**Table 2:** Results of multiple logistic regression of number of ACEs on prescription opioid use during pregnancy and covariates (*N* = 2,999)

Number of ACEs	OR	95% CI
0 (Reference)	—	—
1	1.884	(0.976—3.638)
2	1.986	(0.903—4.368)
3 or More	2.437**	(1.319—4.503)

Control variables include: mother’s age, mother’s race/ethnicity, mother’s educational attainment, currently married, number of prior births, Medicaid, income, state of residence, and year of birth

*Abbreviations:* *OR* Odds ratio, *CI* Confidence interval, *ACEs* Adverse childhood experiences

^**^p < .01

### Supplemental analysis

We conducted a few additional analyses to assess the overall findings. First, considering findings that the use of alcohol, tobacco, and marijuana is associated with prescription opioid use during pregnancy [36, 37], Additional file 2: Appendix D reassesses the main results while controlling for other types of substance use during pregnancy. This analysis includes variables for the average number of *cigarettes* a mother reported smoking per day across all three trimesters of pregnancy (0, less than 10, or 10 or more), the number of *alcoholic drinks* a mother reported consuming per week in the 3 months before becoming pregnancy (0 drinks, less than 1 drink, 1–3 drinks, 4–7 drinks, more than 7 drinks), and whether the mother reported using *marijuana* during pregnancy (1 = yes; 0 = no). After including these control variables, the results from the multiple regression model (*n* = 2,954) report similar results to the main analysis, as respondents with three or more ACEs have significantly higher odds of reporting prescription opioid use during pregnancy relative to respondents with no ACEs (OR = 2.221, 95% CI = 1.162, 4.245).

Next, we conducted a descriptive supplemental analysis using a measure of patterns of prescription opioid use, including *no prescription opioid use*, *pain management*, and *prescription opioid misuse* across ACEs exposure. A description of variable coding is provided in the methodological appendix. Additional file 2: Appendix E shows the percentage of respondents with no opioid use, opioid use for pain management, and opioid misuse by ACEs level. Overall, opioid use for pain management is lowest among respondents with 0 ACEs (2.2%) and raises alongside greater ACEs exposure: 1 ACE = 4.3%, 2 ACEs = 4.5%, 3 + ACEs = 5.6%. A similar pattern is found with pain management, which is reported by 2.1% of respondents with 0 ACE exposure compared to 5.6% with three or more ACEs. Likewise, opioid misuse occurs in 0.1% of individuals with 0 ACEs exposure but 1.9% of persons with three or more ACEs. Results from Fisher’s exact test for *r* X *c* tables determine that the difference between all three types of patterns of opioid use by ACEs level is statistically significant at the *p*< 0.001 level [38, 39]. However, small cell sizes prohibited multivariable analyses, and thus the differences should be interpreted cautiously.

## Discussion

The core findings of this study reveal that ACEs—especially three or more ACEs—were associated with a 2.4-fold increase in the odds of prescription opioid use during pregnancy. These findings confirm earlier research that found a connection between ACEs and prescription opioid use among non-pregnant populations [23, 40, 41]. However, the results differ from recent work conducted by Osofsky et al. [30], which found no association between more ACEs and prescription opioid use for nonmedical reasons during pregnancy among a sample of 303 pregnant women embedded in a psychosocial perinatal support program in a Southern urban medical clinic. In addition, it is important to note that beyond the impact of cumulative ACEs, the current study found that eight out of the 10 ACEs were associated with prescription opioid use during pregnancy. Previous research by Osofsky et al. [30] found a relationship between indicators of child maltreatment and prescription opioid use for nonmedical reasons during pregnancy but no relationship with household dysfunction (i.e., parental separation or divorce, domestic violence, household substance abuse, family mental illness, and family member imprisonment). Somewhat consistent with this finding, our measures of abuse and neglect were consistently associated with prescription opioid use. In contrast, parental separation or divorce and domestic violence (i.e., indicators of household dysfunction) were not. Even so, our findings did indicate that measures related to household mental health, substance use, and incarceration were significantly associated with prescription opioid use. Still, due to differences in samples and because Osofsky et al. focused on prescription opioid use for nonmedical reasons, it is challenging to compare the results of the two studies directly. The findings of this study expand upon prior research by offering critical evidence that the relationship between ACEs and prescription opioid use in adulthood extends to the prenatal period. The finding of elevated patterns of prescription opioid use during pregnancy among women who experienced three or more ACEs offers an essential insight into the enduring role of early life adversity on health behaviors during pregnancy [42]. It is also important to note that most women—even in the face of ACEs exposure—did not use prescription opioids during pregnancy. For instance, while 7.5% of women with three or more ACEs used prescription opioids during pregnancy, over 90% of respondents with high ACEs exposure did not use prescription opioids during this period. Accordingly, while ACEs increased the risk of prenatal prescription opioid use, this was not the case for most pregnant women, suggesting that high levels of early life adversity increase the risk of, but do not guarantee, compromised behavioral health during pregnancy.

The findings from this study contribute to the existing literature regarding how ACEs are adverse outcomes in and of themselves. ACEs often contribute to downstream health effects due to allostatic load and stress and increase the risk of poor health behaviors such as substance use [10–12]. Importantly, the findings in this study offer a key contribution to the literature’s understanding of the consequences of ACEs by demonstrating their connection to the use of prescription opioids during the critical period of pregnancy. The results suggest that even distal life events in childhood and adolescence can shape health behaviors during pregnancy by contributing to increased use of prescription opioids that potentially harm infant health [3–7]. Accordingly, these findings reveal that the consequences of ACEs may be intergenerational, impacting not only the individual directly exposed to ACEs but also potentially harming the health of offspring from the earliest days of life.

While the current study established a connection between ACEs and prescription opioid use during pregnancy, future research should explore potential pathways to explain this relationship. For instance, many recent studies have documented that ACEs are strongly associated with chronic pain in children and adults [43–47], which may lead to prescription opioid use for pain management but could also lead to patterns of opioid misuse. Likewise, ACEs can result in challenges with emotional regulation and a propensity for outlets to alleviate adverse emotional states [25, 48]. Given that pregnancy is an emotionally vulnerable period, such states may be heightened during times of pregnancy, thereby amplifying the risk of prescription opioid use. Uncovering the mechanisms of why ACE exposure leads to an increased risk of prescription opioid use during pregnancy for some women is crucial for developing programmatic interventions to provide support to ACEs exposed populations and promote positive health behaviors and healthy pregnancy.

The results also highlight the importance of detecting and mitigating ACEs’ negative repercussions on behavioral health. One means may be using clinical screenings during prenatal care and primary care visits to detect the presence of ACEs better and, when detected, provide trauma-informed care to help ensure a healthy pregnancy. For instance, recent evidence from two pilot studies in the Kaiser-Permanente system [49] found ACEs screenings can be feasibly conducted in a prenatal care setting without re-traumatization [50], and such practices can improve women’s health outcomes and children. Therefore, assessing the feasibility of such approaches to mitigate prescription opioid use during pregnancy is an important area for future research to consider carefully.

### Limitations

There are limitations to the current analysis that can be expanded upon in future research. First, North Dakota and South Dakota were the only two states which asked questions about ACEs and prescription opioid use during pregnancy in the PRAMS study. Accordingly, the results may not be generalizable outside of these contexts, especially considering that these two states are unique in many regards, such as being more rural and having higher populations of White and Native American persons than the U.S. general population. Second, the questions about ACEs and prescription opioid use may be subject to recall or social desirability bias. Third, the study focused on various prescription opioids used during pregnancy. However, the findings of this study cannot be generalized to the use of illicit non-prescription opioids such as heroin. Fourth, because of sample limitations, we could not examine other questions about opioid use more granularly defined in multivariable analyses, such as whether opioids were used for pain management or nonmedical reasons, the frequency of prescription opioid use, and the timing in the pregnancy during which prescription opioids were used, and from where a respondent obtained the prescription opioids [9]. Larger-scale quantitative studies and qualitative research would be helpful to ascertain better the relationship between ACEs and specific reasons for and patterns of prescription opioid use during pregnancy. Finally, because the PRAMS data are cross-sectional, the findings should be interpreted as associations rather than causal relationships.

## Conclusion

ACEs and prescription opioid uses are serious public health concerns that can influence maternal and infant health. The current study offered a novel insight into the relationship between ACEs and prescription opioid use during pregnancy. The findings suggest the need for additional research to understand better the mechanisms that lead to a link between ACEs and prescription opioid use during pregnancy, as well as how to support those with ACEs exposure in a trauma-informed manner to reduce the risk of subsequent substance use.

### Supplementary Information

**Additional file 1.** Methodological Appendix.

**Additional file 2:** **Appendix A.** Flowchart of Sample Selection Procedure. **Appendix B.** Definition of Adverse Childhood Experiences (ACEs). **Appendix C.** Results of Multiple Logistic Regression of Specific ACEs on Prescription Opioid Use During Pregnancy and Covariates (*N *= 2,999). **Appendix D.** Results of Multiple Logistic Regression of Number of ACEs on Prescription Opioid Use During Pregnancy and Covariates (*N *= 2,999). **Appendix E.** Patterns of Prescription Opioid Use During Pregnancy by ACEs (*N *= 2,999).

## Data Availability

The datasets generated and/or analyzed during the current study are not publicly available due to the nature of Pregnancy Risk Assessment Monitoring System not being publicly available. Data used in this study can be requested at https://www.cdc.gov/prams/index.htm. Queries about the data can be directed to Alexander Testa: alexander.testa@uth.tmc.edu.
