# Supplementary material for: Adverse childhood experiences and prescription opioid use during pregnancy: an analysis of the North and South Dakota PRAMS, 2019–2020
Source: BMC Pregnancy Childbirth. 2023 Aug 23;23:602. doi: 10.1186/s12884-023-05925-7 (PMC10463492; doi:10.1186/s12884-023-05925-7)
Supplement: Supplementary file 1 — Additional file 1. Methodological Appendix. [file 12884_2023_5925_MOESM1_ESM.docx]

**Methodological Appendix**

*Patterns of Opioid Use*

In 2019 and 2020, a subset of women who reported using opioids during pregnancy were asked follow-up questions regarding the source of opioid prescription and reason for use. The source of prescription pain relievers is based on a question asking, "Where did you get the prescription pain relievers that you used during your most recent pregnancy?" (a) OB-GYN, midwife, or prenatal care provider, (b) Family doctor or primary care doctor, (c) Dentist or oral health care provider, (d) Doctor in the emergency room, (e) I had pain relievers left over from an old prescription, (f) Friend or family member gave them to me, (g) I got the pain relievers without a prescription some other way, and (h) Other. The reasons for using opioids were based on a question asking, "What were your reasons for using prescription pain relievers during your most recent pregnancy?" (a) To relieve pain from an injury, condition, or surgery I had before pregnancy, (b) To relieve pain from an injury, condition, or surgery that happened during my pregnancy, (c) To relax or relieve tension or stress, (d) To help me with my feelings or emotions, (e) To help me sleep, (f) To feel good or get high, (g) Because I was "hooked" or had to have them, or (h) Other.

Based on responses to these two questions, women were then categorized into three categories classifying patterns of opioid use: (1) no opioid use includes women who did not use prescription opioids during pregnancy, (2) pain management includes women who received opioids from a health care provider and used opioids to relieve pain, and (3) opioid misuse is defined as getting opioids from a source other than a health care provider or using opioids for a reason other than pain. Consistent with prior research,^1,2^ the qualitative responses to the "other" categories were analyzed, and women were reclassified into the opioid misuse category in cases where a respondent indicated obtaining opioids from a non-healthcare provider (i.e., "off the streets") or used opioids for a reason other than pain relief (i.e., "help me sleep").

**Reference**

1. Ko JY, D’Angelo DV, Haight SC, et al. Vital Signs: Prescription Opioid Pain Reliever Use During Pregnancy — 34 U.S. Jurisdictions, 2019. *Morb Mortal Wkly Rep*. 2020;69(28):897-903. doi:10.15585/mmwr.mm6928a1

2. Testa A, Crawford AD, Jackson DB, Gemmill A. Stressful life events and prescription opioid use during pregnancy: findings from the 2019 pregnancy risk assessment monitoring system. *Soc Psychiatry Psychiatr Epidemiol*. Published online August 10, 2022. doi:10.1007/s00127-022-02349-0
