# Supplementary material for: Adverse childhood experiences and prescription opioid use during pregnancy: an analysis of the North and South Dakota PRAMS, 2019–2020
Source: BMC Pregnancy Childbirth. 2023 Aug 23;23:602. doi: 10.1186/s12884-023-05925-7 (PMC10463492; doi:10.1186/s12884-023-05925-7)
Supplement: Supplementary file 2 — Additional file 2: Appendix A. Flowchart of Sample Selection Procedure. Appendix B. Definition of Adverse Childhood Experiences (ACEs). Appendix C. Results of Multiple Logistic Regression of Specific ACEs on Prescription Opioid Use During Pregnancy and Covariates (N = 2,999). Appendix D. Results of Multiple Logistic Regression of Number of ACEs on Prescription Opioid Use During Pregnancy and Covariates (N = 2,999). Appendix E. Patterns of Prescription Opioid Use During Pregnancy by ACEs (N = 2,999). [file 12884_2023_5925_MOESM2_ESM.docx]

**Appendix A: Flowchart of Sample Selection Procedure**

| Participated in the 2019 or 2020 PRAMS | 45 states  (n = 86, 899) |
| --- | --- |
|  | ↓ |
| Has data on Prescription Opioid Use | 27 states  (n = 35,682) |
|  | ↓ |
| Has data on adverse childhood experiences | 2 states  (n = 3,236) |
|  | ↓ |
| Has data on control variables | 2 states  (n = 2,999) |

**Appendix B: Definition of Adverse Childhood Experiences (ACEs)**

| **ACE Item** | **Definition** | **Prevalence** |
| --- | --- | --- |
| Parents Separated | Were your parents ever separated or divorced? | 38.5% |
| Drinking Problem | Did you live with anyone who was a problem drinker or alcoholic or who used street drugs? | 27.6% |
| Mental Health | Was a household member depressed or mentally ill, or did a household member attempt suicide? | 25.7% |
| Incarceration | Did a household member go to prison? | 10.0% |
| Sexual Abuse | Did an adult or person at least 5 years older than you ever touch or fondle you or have you touch their body in a sexual way OR attempt or actually have oral, anal, or vaginal intercourse with you? | 14.8% |
| Verbal Abuse | Did a parent or other adult in the household swear at you, insult you, put you down, or humiliate you OR act in a way that made you afraid that you might be physically hurt? | 25.8% |
| Physical Abuse | Did a parent or other adult in the household push, grab, slap, or throw something at you OR ever hit you so hard that you had marks or were injured? | 16.2% |
| Emotional Neglect | Did you feel that no one in your family loved you or thought you were important or special OR your family didn’t look out for each other, feel close to each other, or support each other? | 16.1% |
| Physical Neglect | Did you feel that you didn’t have enough to eat, had to wear dirty clothes, and had no one to protect you OR your parents were too drunk or high to take care of you or take you to the doctor if you needed it? | 6.2% |
| Domestic Violence | Was your mother or stepmother pushed, grabbed, slapped, or had something thrown at her OR sometimes, often or very often kicked, bitten, hit with a fist, or hit with something hard OR ever repeatedly hit at least a few minutes or threatened with a gun or knife? | 11.8% |

**Appendix C: Results of Multiple Logistic Regression of Specific ACEs on Prescription Opioid Use During Pregnancy and Covariates (*N* = 2,999)**

| **Number of ACEs** | **OR** | **95% CI** |
| --- | --- | --- |
| Parents Separated | 0.917 | (0.589 - 1.426) |
| Drinking Problem | 1.818** | (1.194 - 2.767) |
| Mental Health | 2.625*** | (1.729 - 3.984) |
| Incarceration | 2.230** | (1.346 - 3.695) |
| Sexual Abuse | 1.845* | (1.150 - 2.961) |
| Verbal Abuse | 1.763** | (1.153 - 2.696) |
| Physical Abuse | 1.836** | (1.169 - 2.882) |
| Emotional Neglect | 1.646* | (1.024 - 2.646) |
| Physical Neglect | 1.807* | (1.016 - 3.212) |
| Domestic Violence | 1.188 | (0.715 - 1.974) |

****p* <.001, ***p* <.01, **p* <.05

Control variables include: mother’s age, mother’s race/ethnicity, mother’s educational attainment, currently married, number of preterm births, Medicaid, income, state of residence, and year of birth

*Abbreviations:* OR = odds ratio; CI = confidence interval, ACEs = adverse childhood experiences

**Appendix D: Results of Multiple Logistic Regression of Number of ACEs on Prescription Opioid Use During Pregnancy and Covariates (*N* = 2,999)**

| **Number of ACEs** | **OR** | **95% CI** |
| --- | --- | --- |
| 0 (Reference) | — | — |
| 1 | 1.850 | (0.942 - 3.633) |
| 2 | 1.890 | (0.824 - 4.334) |
| 3 or More | 2.221* | (1.162 - 4.245) |

***p* <.01

Control variables include: mother’s age, mother’s race/ethnicity, mother’s educational attainment, currently married, number of prior births, Medicaid, income, number of cigarettes smoked during pregnancy, number of alcoholic drinks, marijuana use during pregnancy,

+state of residence, and year of birth

*Abbreviations:* OR = odds ratio; CI = confidence interval, ACEs = adverse childhood experiences

**Appendix E: Patterns of Prescription Opioid Use During Pregnancy by ACEs (*N* = 2,999)**

| **Number of ACEs** | **No Prescription Opioid Use** | **Pain Management** | **Misuse** |
| --- | --- | --- | --- |
| 0 | (1,005) 97.7% | (28) 2.1% | (4) 0.1% |
| 1 | (567) 95.3% | (24) 4.3% | (3) 0.4% |
| 2 | (314) 94.5% | (14) 4.5% | (4) 1.0% |
| 3 or More | (941) 92.5% | (66) 5.6% | (29) 1.9% |

*Note:* Table reports unweighted frequencies in parentheses and weighted percentages.
